# Post Partum Hemorrhage

**Published:** 1877-10-20

**Authors:** J. Morrison

**Affiliations:** Member of the Council and Examiner on Chemistry in the College of Physicians and Surgeons, of Ontario


### POST PARTUM HEMORRHAGE.

By J. Morrison, M.D., A.M., Member of the Council and Examiner on Chemistry in the College of Physicians and Surgeons, of Ontario.

*Treatment*—In a matter of so serious a nature and of such an alarming character, the principles of treatment cannot be too well understood by the practitioner; hence the propriety of frequently calling the attention of the younger members of our profession, especially, to so important a subject. If the pla–

centa has been removed, or if it is partially adherent to the uterus, and the organ in a state of inertia, introduce the hand into the womb and with the back of the fingers make firm and uniform pressure against the bleeding vessels, and with the other hand make counter pressure on the abdomen by friction or kneading. If the organ does not immediately contract, without a moment's delay, dash cold water suddenly on the abdomen, and repeat, should it be necessary. As soon as contraction is felt, the placenta, if not previously delivered, may be grasped with the hand until both are expelled, friction and kneading being applied externally in the meantime. If a portion of the placenta is retained, it must be removed as soon as possible by introducing the hand, if necessary, except when syncope has occurred with the formation of a clot. In such a case complete reaction must be allowed to take place before attempting to remove it. These are the heroic but common sense measures on which we must depend in desperate cases, when it would be all but useless to place our dependence on remedies administered by the mouth. Much benefit may, however, be derived from the patient swallowing small pieces of ice, which by their action on the sympathetic system of nerves often bring on instantaneous contraction, In the external application of cold as a remedy in uterine hemorrhage, we must bear in mind that when it is too long applied it becomes an in-exciter, and is also liable to bring on great prostration and coldness of the extremities. Warmth must then be applied, and some gentle stimulants, such as a little brandy and water, administered, an alternation of heat and cold being in fact one of the most powerful excitors of the spinal cord.

In perilous cases of hemorrhage, however, cold will very seldom have to be applied long enough to lose its effect as an excitor of the uterine nerves, and, therefore, the alternation of heat and cold will be rarely called for.

The position of the patient is also important. The pillows should be removed from under the head, and the hips elevated by raising the foot of the bed. Perfect quiet should also be enjoined—a measure not always attainable under such circumstances. Besides the remedies just mentioned—viz: cold and pressure—there are several others which, though not so prompt and immediate in their action, must not be overlooked. Of these ergot and oil of erigeron canadense hold the first place. One or the other of these medicines should be administered as soon as practicable after the birth of the child, as auxiliary means, and indeed profuse hemorrhage may, perhaps, be always warded off by their timely administration. Ergot has lately been proved to owe its virtues to phosphoric acid, and, it is said, on respectable authority, that the acid alone will accomplish the same results.

The other remedy, viz: the oil of erigeron, is more certain than ergot, and has a far wider range of application, it being one of the most valuable remedies we possess in secondary hemorrhage and menorrhagia.

Entire dependence should never be placed on either ergot or oil of erigeron, as they are by no means instantaneous in their action. They require from five to twenty minutes before their effects become manifest, and in this time either death would ensue or the patient's life be seriously jeopardized. As auxilliary means, however, they are most valuable, and should never be omitted. Both of these remedies will be found more efficacious when administered along with a few drops of tincture of capsicum, or in its absence with a little brandy. In secondary hemorrhage, and, indeed, in all hemorrhages from the uterus, our main reliance may be placed on the oil of erigeron. This is one of the remedies peculiar to our school of medicine, and if we had added no other remedy to the armamentum of the physician, we would have deserved the everlasting gratitude of our race for having enriched the materia medica with a remedy which has no superior in this class of hemorrhages. Notwithstanding its well known power to control both hemorrhage and diarrhœa, it has not come into general use among all classes of practitioners, but like quinia, veratrum viride, and others, it will require about half a century before physicians of all schools will appreciate and acknowledge its virtues. Gossypium is another remedy which is said to possess considerable power in promoting uterine contraction, but from the difficulty in procuring a good preparation from the recent root, it is doubtful whether it will ever take the place of more efficient remedies.

With regard to the injection of astringents and other irritating substances into the uterus in such cases, they will be found to be pernicious in their tendency and uncertain in their action. Electricity, too, though highly lauded by some authorities, cannot be readily applied, owing to the apparatus being rarely at hand, and even if it were, death would occur before it could be applied.

Another remedy which is still in use in some quarters, and is indeed a favorite remedy with those who persist in remaining behind the age—the tampon—deserves to be only mentioned that it may be condemned. It can exercise no power in contracting the uterus, the primary object in view, but, on the contrary, rather retards contraction by preventing the flow of blood through the vagina, and thus converts an external hemorrhage into an internal one, thereby deceiving the practitioner and most certainly destroying the patient.

After the hemorrhage has been controlled, if the patient has lost much blood, she will be found frequently pulseless and with cold extremities—in fact in a state of great prostration. Under such circumstances a little brandy, with or without tincture of opium, may be cautiously administered until the pulse returns and a general warmth to the extremities. These remedies may be also aided by applying warm flannels and bottles filled with warm water to the limbs. As soon as reaction is fully established the stimulants must be withdrawn and the patient's strength sustained by such measures as the general circumstances of the case will indicate. For quieting the nervous system and mitigating the severity of after pains, there is nothing in the materia medica so efficacious as a decoction of scutellaria.

After profuse hemorrhage the patient will almost always complain of more or less headache and intolerance of light. Scutellaria will relieve these promptly.

